# Combination of EGFR-TKIs and Chemotherapy as First-Line Therapy for Advanced NSCLC: A Meta-Analysis

**DOI:** 10.1371/journal.pone.0079000

**Published:** 2013-11-13

**Authors:** Pu-Yun OuYang, Zhen Su, Yan-Ping Mao, Wuguo Deng, Fang-Yun Xie

**Affiliations:** 1 State Key Laboratory of Oncology in South China, Collaborative Innovation Center for Cancer Medicine, Guangzhou, China; 2 Department of Radiation Oncology, Sun Yat-sen University Cancer Center, Guangzhou, China; Hormel Institute, University of Minnesota, United States of America

## Abstract

The impact of combining epidermal growth factor receptor tyrosine kinase inhibitors (EGFR–TKIs) and chemotherapy as first-line therapy for patients with advanced non-small-cell lung cancer (NSCLC) remains controversial. Therefore, randomized trials that compared this combined regimen with chemotherapy or EGFR–TKIs monotherapy were included for this meta-analysis. We used published hazard ratios (HRs), if available, or derived treatment estimates from other survival data. Pooled estimates of treatment efficacy of the combined regimen in the entire unselected population and selected patients by EGFR-mutation status and smoking history were calculated. Eight trials eventually entered into this meta-analysis, including 4585 patients. Overall, the combined regimen significantly delayed disease progression (HR = 0.81, 95% CI 0.69–0.95, *P* = 0.01); subgroup analysis showed significantly higher progression free survival advantages in Asian patients (*P*<0.001), with sequential combination of TKIs and chemotherapy (*P* = 0.02). In selected patients by EGFR-mutation, both mutation positive (HR = 0.48, 95% CI 0.28–0.83, *P* = 0.009) and negative (HR = 0.84, 95% CI 0.72–0.98, *P* = 0.02) patients gained progression free survival benefit from the combined regimen, albeit the magnitude of benefit was marginally larger in mutation positive patients (*P* = 0.05). In selected patients by smoking history, never/light smokers achieved a great progression free survival benefit from the combined regimen (HR = 0.51, 95% CI 0.35–0.74, *P* = 0.0004). Unfortunately, the combined regimen had no significant impact on overall survival, irrespective of ethnicity, dose schedules or EGFR-mutation status. Severe anorexia (RR = 2.01, 95% CI 1.11–3.63; *P* = 0.02) and diarrhea (RR = 2.70, 95% CI 1.94–3.76; *P*<0.001) were more frequent in the combined regimen arm. This strategy of combining EGFR–TKIs and chemotherapy deserved to be considered in the future, although it is not approved for advanced NSCLC at the moment.

## Introduction

For advanced non-small-cell lung cancer (NSCLC) patients, platinum-based chemotherapy was recommended as standard regimen for initial treatment. Advances in genetic testing allowed the discovery and clinical application of driver oncogenes, such as activating epidermal growth factor receptor (EGFR) mutations, as a therapeutic target. Several randomized controlled trials [1]–[4] and meta-analyses [5], [6] have demonstrated that EGFR-tyrosine-kinase inhibitor (EGFR–TKI), erlotinib or gefitinib, is superior to chemotherapy as first-line treatment for patients with EGFR mutations. However, patients may still have unknown EGFR mutation status at the time when first-line treatments decisions are made, due to limited high-quality tumor samples or insufficient testing facilities. Treating patients with unknown EGFR-mutations with a combination of chemotherapy and an EGFR–TKI is an applicable option. Unfortunately, previous randomized trials showed no significant improvement of survival by combining EGFR–TKIs and chemotherapy [7]–[12]. But another phase II trial of sequential combination of erlotinib and chemotherapy as first-line treatment for advanced NSCLC showed a significant improvement in progression-free survival (PFS) [13]. And the interesting findings are recently confirmed in the phase III trial – FASTACT–II [14]. Controversy continues regarding the role of the addition of EGFR–TKIs in patients receiving chemotherapy. Therefore, we conducted this meta-analysis to comprehensively estimate the treatment effect of the combined regimen on PFS and overall survival (OS) based on characteristics of patients.

## Materials and Methods

### Search Strategy and Selection Criteria

All randomized trials evaluating the effect of the combined regimen of EGFR–TKIs and chemotherapy were eligible for inclusion. Two investigators (P. Y. OuYang and Z. Su) independently searched PubMed database, Cochrane Controlled Trials Register via Cochrane Library and ClinicalTrials.gov with the terms “erlotinib OR tarceva”, “gefitinib OR iressa”, “chemotherapy”, “first-line”, and “non-small-cell lung cancer OR NSCLC”. The search was limited to randomized controlled trials or clinical trials. We also searched the conference proceedings of the American Society of Clinical Oncology (ASCO), the European Society of Medical Oncology and the International Association for the Study of Lung Cancer for relevant clinical trials.

Eligible trials satisfying the following requirements were eventually included: (a) prospective randomized controlled trials (phase II or III), (b) chemotherapy-naïve patients with cancer were randomly assigned to first-line treatment with chemotherapy or an EGFR–TKI monotherapy or the combined regimen of EGFR–TKI and chemotherapy, (c) adequate survival data available for calculation or estimation of a hazard ratio (HR) with a 95% confidence interval (CI). Phase I study and phase II study with only one single arm were excluded because of either drug dosage difference or the missing control group.

### Data Extraction and Study Quality Assessment

Two authors (P. Y. OuYang and Z. Su) independently identified eligible trials and extracted information on trial name, year of publication, name and dosage of EGFR–TKI, trial design and treatment protocol, number of patients in investigational and control arms, median age (range), sex (female), race (Asian), never/light smoker and severe toxicities. Mutational analysis data was also extracted. Patients were classified as EGFR mutation–positive if a mutation was detected using molecular assessment tools such as Sanger sequencing, polymerase chain reaction clamp, and amplification refractory mutation system. Patients were classified as EGFR mutation–negative if no mutation was detected. We did not classify the EGFR mutation status based on immunohistochemistry and fluorescent in situ hybridization for EGFR gene copy numbers.

To award study quality, we examined the randomization procedure, estimation of sample size, blinding, loss to follow-up, dropout and if the intention-to-treat analysis was followed.

### Statistical Analysis

We extracted HRs and associated 95% CIs for PFS and OS outcomes to assess treatment efficacy. If HR and CI were not reported, these were estimated using the methods of Parmar [15]. Risk ratio (RR) with 95% CI was used for results of comparing severe toxicities in both arms.

Heterogeneity across studies was estimated by Chi-square test and I^2^ statistic and correct effects models were chosen accordingly. Statistically significant heterogeneity was defined as a Chi-square *P* value less than 0.1 or an I^2^ statistic greater than 50% [16]. If heterogeneity was not observed, we just reported the summary estimation results on the basis of fixed-effects model. If heterogeneity was observed, the summary estimation was based on random-effects model. Subgroup analysis was conducted to detect evident heterogeneities.

Potential publication bias was assessed with the Begg’s test and Egger’s test, and graphically presented by funnel plots. All statistical analysis was performed by Review Manager Version 5.2 (Revman; the Cochrane Collaboration; Oxford, England) and STATA version12.0. A two-sided *P* value of less than 0.05 was considered significant for all analysis except heterogeneity tests.

## Results

### Eligible Studies

**Figure 1:**
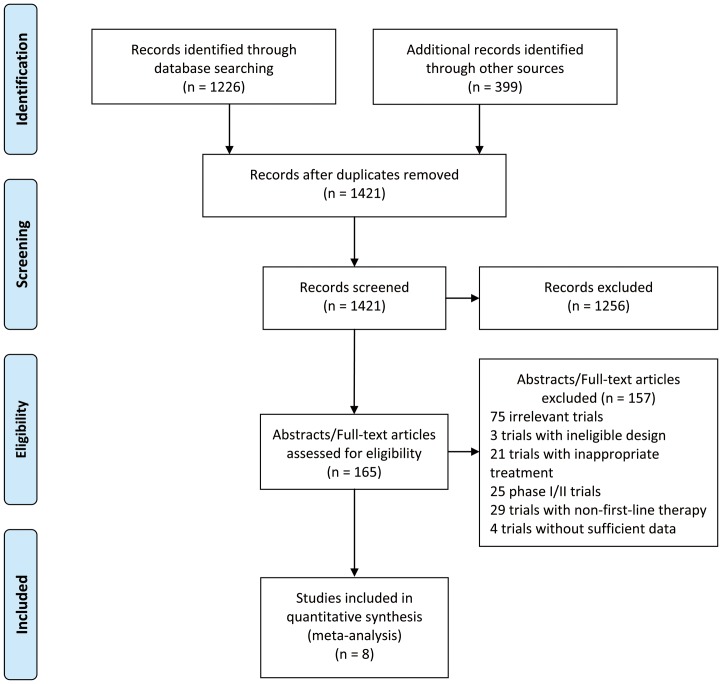
Flow diagram of identifying trials.

Overall, eight trials [7]–[14] were highly eligible for inclusion in this meta-analysis (Figure 1). Six trials (INTACT 1 [7], INTACT 2 [8], TALENT [9], TRIBUTE [10], FASTACT [13] and FASTACT–II [14]) compared the combined regimen with chemotherapy alone, while the other two trials (trial by Hirsch et al [11] and CALGB 30406 trial [12]) compared this combination with EGFR–TKIs monotherapy. Participants in the FASTACT [13], FASTACT–II [14] and trial by Hirsch et al [11] were administered with platinum-based chemotherapy sequentially followed by erlotinib or placebo, whereas patients in the other trials were delivered with concurrent dosing schedules. The baseline characteristics of ethnicity, adenocarcinoma histology, never/light smoking history, female gender and EGFR mutation were presented in Table 1. However, survival information was only available in selected patients by smoking history and EGFR mutation status.

**Table 1:** Baseline characteristics of the included trials in the meta-analysis.

Trials(year)	TKIs	chemotherapy (dose*cycles)	Patientsanalyzed	Median age(range)	Female	Race (% Asian)	Never/light smoker	EGFR mutation positive
FASTACT(2009) [13]	E†	DDP(75 mg/m^2^,d1)/CBP(AUC = 5,d1)+GEM1250(mg/m^2^,d1,8),q4w*6	76vs78	57.5(33–79) vs57.0(27–79)	22vs24	93vs95	24vs28	2vs5
FASTACT–II (2013) [14]	E†	DDP(75 mg/m^2^,d1)/CBP(AUC = 5,d1)+GEM1250(mg/m^2^,d1,8),q4w*6	226vs225	59.0(31–96)vs57.3(37–88)	94vs85	100vs100	112vs107	49vs48
INTACT 1(2004) [7] [**17**]	G‡	DDP(80 mg/m^2^,d1)+GEM(1250 mg/m^2^d1,8),q3w*6	365vs363	59(34–83)vs61(33–81)	85vs101	1.6vs0.8	NA	23vs9&
INTACT 2(2004) [8] [**17**]	G‡	CBP(AUC = 6)+TAX(225 mg/m^2^),q3w*6	345vs345	61(27–86)vs63(31–85)	146vs133	NA	NA	
TALENT(2007) [9]	E	DDP(80 mg/m^2^,d1)+GEM(1250 mg/m^2^d1,8),q3w*6	580vs579	61(26–82)vs60(28–84)	125vs142	3vs4	8vs10	NA
TRIBUTE(2005) [10] [**18**]	E	CBP(AUC = 6)+TAX(200 mg/m^2^),q3w*6	539vs540	63(24–84)vs63(26–84)	217vs207	3.9vs2.4	72vs44	15vs14
CALGB30406(2012) [12]	E	CBP(AUC = 6)+TAX(200 mg/m^2^),q3w*6	100vs81	60(34–81)vs58(32–78)	58vs49	8vs6	100vs81	33vs33
Hirsch et al.2011 [11]	E	CBP(AUC = 6)+TAX(200 mg/m^2^),q3w*4	71vs72	NA	31vs44	6vs12	NA	6vs9

Note: TKIs = tyrosine kinase inhibitors, PS = performance status, E = erlotinib, G = gefitinib, DDP = cisplatin, CBP = carboplatin, AUC = area under the curve, GEM = gemcitabine, q4w = every four weeks, vs = the combined regimen versus chemotherapy or TKIs monotherapy, NA = not available, TAX = paclitaxel.

† Sequential administration of erlotinib following gemcitabine/platinum chemotherapy, rather than concurrent administration as the other trials.

‡ Only included patients treated with gefitinib 250 mg/d.

& Data from trials INTACT 1and 2 together.

Overall, these studies were of high quality – blinding, showing randomization procedure, conducting estimation of sample size, mostly reporting dropout and following the principle of intention-to-treat analysis. (Table S1).

### Effect of the Combined Regimen on PFS and OS in Unselected Patients

**Figure 2:**
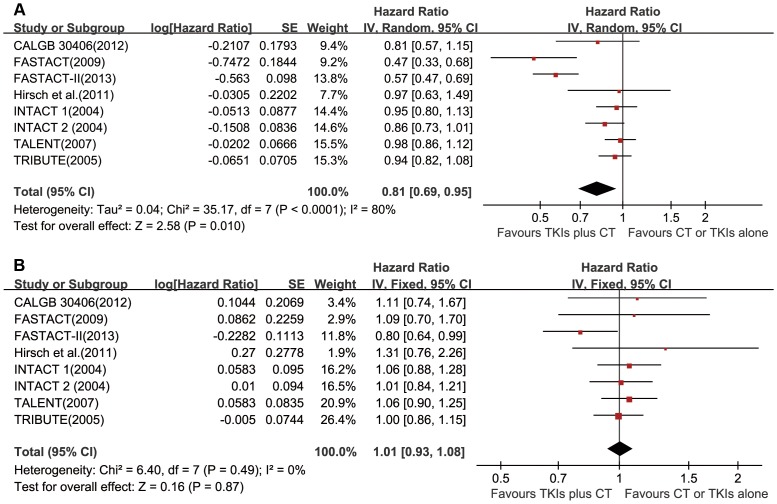
Forest plots in unselected patients. HRs and 95% CIs of (a) progression-free survival and (b) overall survival. TKIs = tyrosine kinase inhibitors, CT = chemotherapy, SE = standard error.

Data of four trials [9], [10], [13], [14] was directly available, while the information was estimated from survival curves in the other trials. Significant PFS benefit was observed from the combined regimen of TKIs and chemotherapy (HR = 0.81, 95% CI 0.69–0.95, *P* = 0.01; Figure 2a) based on random-effects model, due to significant heterogeneity (Chi^2^ = 35.17, *P*<0.001; I^2^ = 80%). Unfortunately, there was no evidence of improvement in OS with the combined regimen (HR = 1.01, 95% CI 0.93–1.08, *P* = 0.87, fixed-effects model; Figure 2b).

Subgroup analysis was conducted according to the regimen in the control group, ethnicity and dose schedules (Table 2). Significant associations between PFS improvement and ethnicity or dose schedules were observed. There were significant higher PFS advantages in Asian patients (*P*<0.001), with sequential combination of TKIs and chemotherapy (*P* = 0.02). Interestingly, the combined regimen was superior over chemotherapy alone in PFS (*P* = 0.01), whereas it was similar to TKIs monotherapy (*P* = 0.32). However, this discrepancy was non-significant (*P* = 0.58), which may be caused by relatively small number of patients in the subgroup of the combined regimen versus TKIs monotherapy. As observed in the entire unselected population, there was no significant OS improvement in subgroups.

**Table 2:** Subgroup analysis in unselected patients.

Subgroup	Included trials	HR (95%CI)	*P* values for heterogeneity	HR (95%CI)	*P* values for heterogeneity
**Regimen in control**					
TKIs	Hirsch [11] and CALGB 30406 [12]	0.87 [0.66, 1.14]	0.58	1.18 [0.85, 1.63]	0.33
Chemotherapy	INTACT 1 [7], INTACT 2 [8], TALENT [9],TRIBUTE [10], FASTACT [13], FASTACT–II [14]	0.79 [0.66, 0.96]		1.00 [0.92, 1.08]	
**Ethnicity**					
Asian	FASTACT [13] and FASTACT–II [14]	0.55 [0.46, 0.65]	<0.001	0.85 [0.70, 1.03]	0.06
Non-Asian	INTACT 1 [7], INTACT 2 [8], TALENT [9],TRIBUTE [10], Hirsch [11], CALGB 30406 [12]	0.93 [0.87, 1.00]		1.04 [0.96, 1.12]	
**Dose schedule**					
Sequential	Hirsch [11], FASTACT [13], FASTACT–II [14]	0.62 [0.44, 0.87]	0.02	0.89 [0.74, 1.07]	0.15
Concurrent	INTACT 1 [7], INTACT 2 [8], TALENT [9],TRIBUTE [10], CALGB 30406 [12]	0.93 [0.87, 1.00]		1.03 [0.95, 1.12]	

### Effect of the Combined Regimen on PFS and OS in Selected Patients by EGFR-Mutation Status

**Figure 3:**
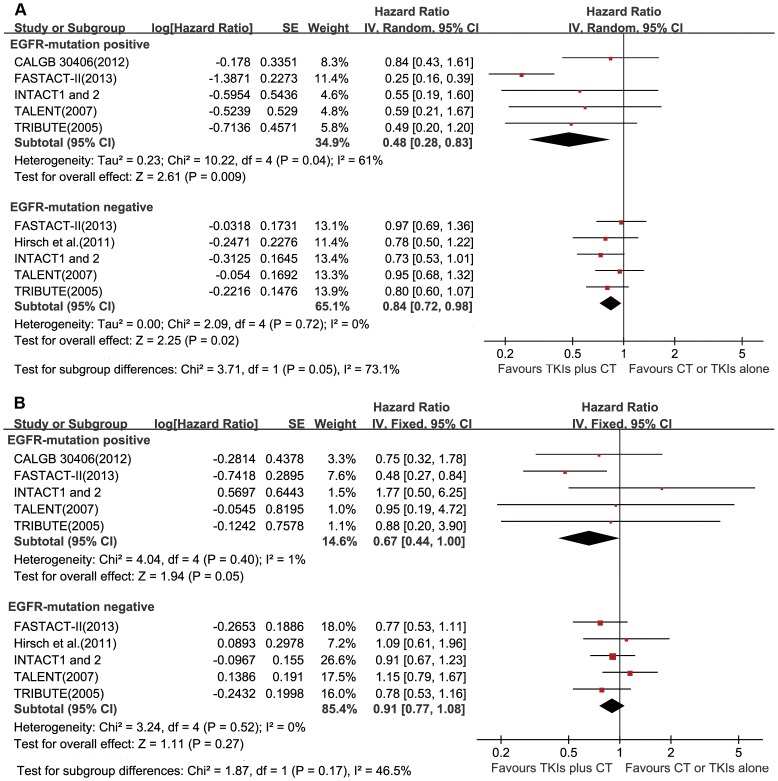
Forest plots in selected patients by EGFR-mutation status. HRs and 95% CIs of (a) progression-free survival and (b) overall survival. TKIs = tyrosine kinase inhibitors, CT = chemotherapy, SE = standard error.

Survival data of EGFR-mutation positive patients was only available in the FASTACT–II [14], INTACT 1 and 2 [17], TALENT [9], TRIBUTE [18] and CALGB30406 [12]. Estimates of PFS and OS in EGFR-mutation negative patients could only be calculated in the FASTACT–II [14], INTACT 1 and 2 [17], TALENT [9], TRIBUTE [18] and trial by Hirsch et al [11]. In the EGFR-mutation positive cohort, the combined regimen was superior over chemotherapy or TKIs monotherapy with a significant improvement in PFS (HR = 0.48, 95% CI 0.28–0.83, *P* = 0.009; Figure 3a). Interestingly, the combined regimen also showed significant PFS benefit in the EGFR-mutation negative cohort, compared with chemotherapy or TKIs monotherapy (HR = 0.84, 95% CI 0.72–0.98, *P* = 0.02; Figure 3a). Certainly, the magnitude of PFS improvement resulted from the combined regimen in the EGFR-mutation positive cohort was marginally larger than that in the EGFR-mutation negative cohort (*P* = 0.05).

In terms of OS, the combined regimen marginally enhanced OS of EGFR-mutation positive patients (HR = 0.67, 95% CI 0.44–1.00, *P* = 0.05), but not EGFR-mutation negative patients (HR = 0.91, 95% CI 0.77–1.08, *P* = 0.27). (Figure 3b).

### Effect of the Combined Regimen on PFS in Never/light Smokers

**Figure 4:**
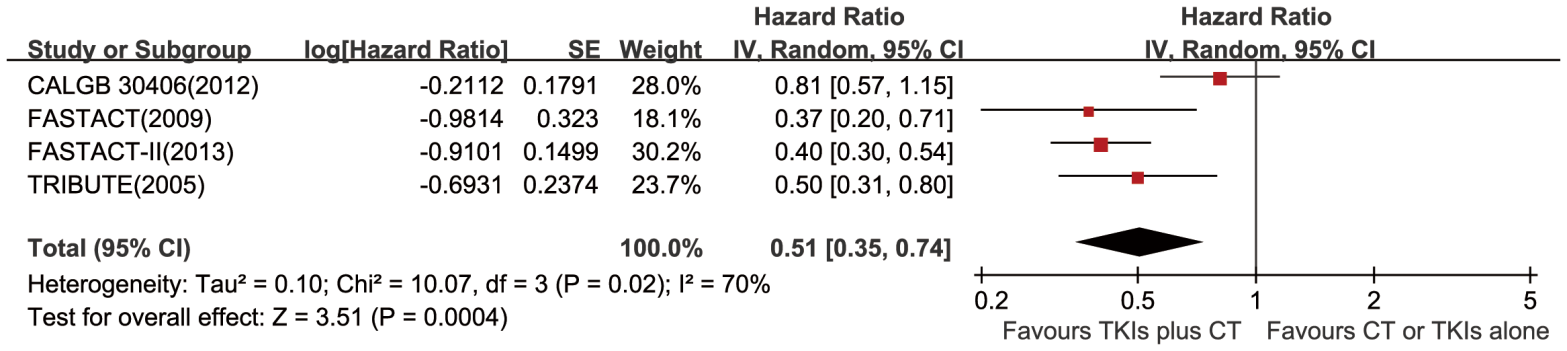
Forest plots in never/light smokers. HRs and 95% CIs of progression-free survival. TKIs = tyrosine kinase inhibitors, CT = chemotherapy, SE = standard error.

We pooled analysis of FASTACT [13], FASTACT–II [14], TRIBUTE [10] and CALGB30406 [12], and found an improvement of PFS in never/light smokers with the combined regimen (HR = 0.51, 95% CI 0.35–0.74, *P* = 0.0004). (Figure 4).

### Grade 3 and Higher Toxicities

Compared with chemotherapy or TKIs monotherapy, the combined regimen caused more grade 3 and higher anorexia (RR = 2.01, 95% CI 1.11–3.63; *P* = 0.02) and diarrhea (RR = 2.70, 95% CI 1.94–3.76; *P*<0.001). And there were no differences of other severe toxicities between the two arms. (Table 3).

**Table 3:** Grade 3 and higher toxicities by meta-analysis for the combined regimen versus chemotherapy or TKI monotherapy.

Subgroup	Trials with data	Risk ratio (95%CI)	*P* values
**Hematologic**			
Anemia	INTACT 1 [7], INTACT 2 [8], TALENT [9], TRIBUTE [10], FASTACT [13],FASTACT–II [14] and CALGB 30406 [12]	0.98 [0.63, 1.53]	0.93
Leukopenia	INTACT 1 [7], INTACT 2 [8], TALENT [9], TRIBUTE [10] and FASTACT–II [14]	0.97 [0.74, 1.27]	0.84
Thrombocytopenia	INTACT 1 [7], TALENT [9], TRIBUTE [10], CALGB 30406 [12],FASTACT [13] and FASTACT–II [14]	1.15 [0.93, 1.41]	0.20
Neutropenia	INTACT 1 [7], INTACT 2 [8], TALENT [9], TRIBUTE [10], Hirsch [11],CALGB 30406 [12], FASTACT [13] and FASTACT–II [14]	1.23 [0.88, 1.73]	0.23†
**Non-hematologic**			
Rash	INTACT 1 [7], INTACT 2 [8], TALENT [9], TRIBUTE [10], Hirsch [11],CALGB 30406 [12], FASTACT [13] and FASTACT–II [14]	2.08 [0.60, 7.16]	0.25†
Nausea	INTACT 1 [7], INTACT 2 [8], TALENT [9], TRIBUTE [10], Hirsch [11],CALGB 30406 [12], FASTACT [13] and FASTACT–II [14]	0.95 [0.40, 2.23]	0.90†
Vomiting	INTACT 1 [7], INTACT 2 [8], TALENT [9], TRIBUTE [10], Hirsch [11],CALGB 30406 [12], FASTACT [13] and FASTACT–II [14]	1.09 [0.81, 1.48]	0.57
Anorexia	INTACT 1 [7], INTACT 2 [8], TALENT [9], Hirsch [11] and FASTACT [13]	2.01 [1.11, 3.63]	0.02
Fatigue/Asthenia	INTACT 1 [7], INTACT 2 [8], TALENT [9], TRIBUTE [10], Hirsch [11],CALGB 30406 [12], FASTACT [13] and FASTACT–II [14]	1.53 [0.78, 2.99]	0.21†
Diarrhea	INTACT 1 [7], INTACT 2 [8], TALENT [9], TRIBUTE [10], Hirsch [11],CALGB 30406 [12], FASTACT [13] and FASTACT–II [14]	2.70 [1.94, 3.76]	<0.001
Dyspnea	INTACT 2 [8], TALENT [9], TRIBUTE [10], and FASTACT–II [14]	0.88 [0.62, 1.23]	0.45

† Using random-effects model for heterogeneity.

### Publication Bias

No publication bias was observed in the meta-analysis (Begg’s test *P*≥0.108, Egger’s test *P*≥0.134). We showed funnel plot of PFS in unselected patients (Figure S1).

## Discussion

Petrelli et al [19] in their meta-analysis collected data of patients with EGFR-mutation from INTACT 1, INTACT 2, TRIBUTE and other 10 trials, and found that NSCLCs harboring EGFR mutations derived greater benefit from erlotinib or gefltinib than from chemotherapy; however, they did not include data from the most recent trials [13], [14], and main results of OS and PFS were based on all trials irrespective of the line of treatment. Another recent meta-analysis [20] compared TKIs plus platinum-based doublet chemotherapy (PBDC) with PBDC alone, and showed marginally improved PFS from the combined regimen; but importantly, it did not explore the effect in selected patients by EGFR-mutation status or demographic factors, nor did it compare survival differences in subgroups according to ethnicity or dose schedules of TKIs and chemotherapy.

Single agent of EGFR–TKIs, either erlotinib or gefitinib, has been demonstrated to be superior to chemotherapy [2]–[6], [13] and recommended by NCCN guideline for first-line treatment of EGFR-mutation positive patients. But it is common that the EGFR-mutation status of the majority of patients is still unknown at the time of making a first-line treatment decision. This meta-analysis incorporates results of eight trials in nearly 4600 patients, and supports the point that combining EGFR–TKIs and chemotherapy is superior in delaying disease progression for advanced NSCLC. The ethnicity and dose schedules of TKIs and chemotherapy greatly influenced the efficacy of the combined regimen in PFS. The magnitude of PFS benefit was larger for the Asian patients, with sequential administration of TKIs and chemotherapy. This study also showed that EGFR mutation was an important predictive biomarker of treatment benefit in terms of PFS. The magnitude of PFS benefit was not similar between EGFR-mutation positive and negative subgroups, although the combined regimen showed significant improvement in both.

As we know, somatic mutations in the EGFR kinase domain had been discovered in a subset of NSCLC [21]–[25]. The two most frequent mutations were the exon 19 deletion that removed residues 746–750 of the expressed protein and the exon 19 point substitution that replaced leucine 858 with arginine (L858R) [24], [25]. Structurally, these mutations clustered around the active site cleft of the tyrosine kinase domain. Comparison of the structures of the mutant kinases with the inactive wild-type EGFR indicated that the mutations were expected to destabilize the inactive conformation, and therefore to promote the active conformation of the kinase. In particular, the L858R mutation was clearly incompatible with the inactive conformation. [26]. Direct measurement of the binding affinity of gefitinib to the wild type and mutant kinases revealed that gefitinib binds the L858R mutant 20-fold more tightly than the wild-type kinase [26]. Based on the kinetics of the wild type and mutant kinases in vitro, the L858R mutant was 50-fold more active than the wild-type kinase [26]. Additionally, cells bearing the mutant EGFR were in general more sensitive to EGFR–TKIs than cells expressing the wild type kinase. The L858R mutant was 10–100 fold more sensitive to erlotinib and gefitinib than the wild type kinase [23], [27]. These basic researches, randomized controlled clinical trials [1]–[4] and meta-analyses [5], [6] thoroughly demonstrated the better treatment outcomes of the EGFR–TKIs in mutation positive patients. Moreover, preclinical studies [28], [29] had clarified a synergistic effect of combining EGFR–TKIs with chemotherapy. Therefore, it is not unexpected that the combined therapy of EGFR–TKIs and chemotherapy shows higher benefit in EGFR-mutation positive cohort.

Remarkably, erlotinib and chemotherapy such as gemcitabine/cisplatin had different mechanisms of action (cytostatic and cytotoxic, respectively). The antiproliferative effects of erlotinib, arising from cell-cycle arrest [30], might render tumor cells less sensitive to cytotoxic agents, as suggested by recent preclinical studies of combinations of EGFR TKIs with chemotherapy [31], [32]. That is, the concurrent schedule might have the potential issue of cell cycle–based antagonism between TKIs and chemotherapy, while the special combination of sequential administration of erlotinib following chemotherapy in FASTACT [13] and FASTACT–II [14] appeared to be successful in that respect, and led to a significant improvement in PFS. Furthermore, considering the heterogeneity of EGFR-mutation in intratumor tissue [33], the sequential schedule had its advantage in theory. When comparing sequential dose schedules with concurrent, we did observe significant differences in effects (*P* = 0.02).

In spite of a large PFS benefit, this meta-analysis did not demonstrate OS advantage with the combined regimen. Regardless of EGFR-mutation status, the overall treatment effects on OS were similar. Limited number of patients with mutational analysis possibly underpowered the effect of the combined regimen in EGFR-mutation positive patients. Additionally, appropriate dose schedules of the combined regimen might contribute to OS benefit, as OS benefit was noted in EGFR-mutation positive patients with sequential combination of chemotherapy and erlotinib in a single trial – the FASTACT–II [14]. More importantly, differences in OS are potentially affected not only by treatment allocation, but also by differences of second or third-line treatment given to patients in both arms after disease progression. A recent systematic review of chemotherapy trials indicated that PFS advantage was unlikely to be associated with OS advantage with increasing impact of salvage therapy and that the prolongation of survival postprogression might limit the role of OS for assessing true efficacy derived from front-line therapy [34].

This meta-analysis had several limitations. Firstly, all data was extracted from published studies, which might result in publication bias and selection bias. Secondly, EGFR-mutation status was only assessed in approximately 20% patients enrolled in eligible trials, with treatment efficacy estimated from small numbers of EGFR-mutation positive patients identified in many of these trials. The potential influence on the results of restricting our analysis to this subset of patients is unknown. Additionally, albeit evidences showed that the distinct EGFR mutations could differ markedly in their EGFR–TKIs susceptibilities [26], [35], [36], it was difficult to perform stratification analysis by the EGFR mutations due to absence of original data and small sample size in respective strata. Thirdly, we performed analysis of ethnicity on the assumption that all patients in FASTACT and FASTACT–II [13], [14] were Asian, while the others were non-Asian according to the actual percent of race (Table 1).

In conclusion, on the basis of this meta-analysis, combination of EGFR–TKIs and chemotherapy leads to PFS benefit as first-line treatment for advanced NSCLC, regardless of EGFR-mutation status, but has no demonstrable impact on OS. And there is a larger magnitude of PFS benefit for Asian patients, with sequential administration of EGFR–TKIs and chemotherapy. EGFR-mutation status is still a predictive biomarker of benefit with the combined regimen, for a larger magnitude of improvement in EGFR-mutation positive patients. This strategy deserved to be considered in the future although it is not approved for advanced NSCLC at the moment.

## Supporting Information

Figure S1 **Funnel plot of progression-free survival of unselected patients.** TKIs = tyrosine kinase inhibitors, CT = chemotherapy, SE = standard error.
(TIF)

Table S1 **Quality assessment of the included studies.**
(DOCX)

Checklist S1 **PRISMA checklist.**
(DOC)
