# Supplementary figures and images for: Combination of EGFR-TKIs and Chemotherapy as First-Line Therapy for Advanced NSCLC: A Meta-Analysis
Source: PLoS One. 2013 Nov 13;8(11):e79000. doi: 10.1371/journal.pone.0079000 (PMC3827342; doi:10.1371/journal.pone.0079000)

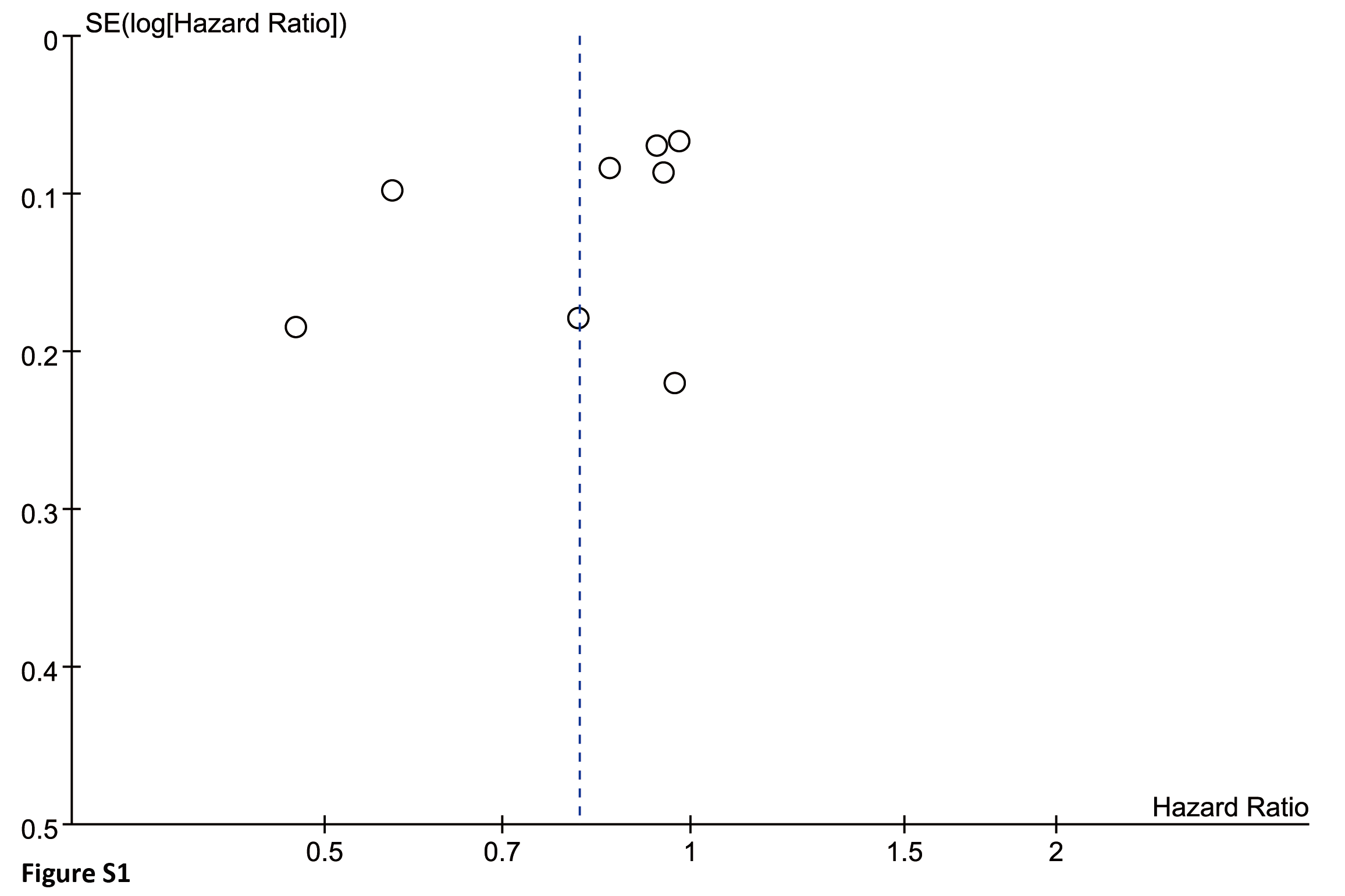

Supplement: Figure S1 — Funnel plot of progression-free survival of unselected patients. TKIs = tyrosine kinase inhibitors, CT = chemotherapy, SE = standard error. (TIF) [file pone.0079000.s001.tif]
