# Supplementary material for: Combination of EGFR-TKIs and Chemotherapy as First-Line Therapy for Advanced NSCLC: A Meta-Analysis
Source: PLoS One. 2013 Nov 13;8(11):e79000. doi: 10.1371/journal.pone.0079000 (PMC3827342; doi:10.1371/journal.pone.0079000)
Supplement: Table S1 — Quality assessment of the included studies. (DOCX) [file pone.0079000.s002.docx]

**Table S1**. Quality assessment of the included studies

| Studies | Randomization procedure | Estimation of sample size | blinding | Loss to follow-up | Intention to treat analysis | dropout |
| --- | --- | --- | --- | --- | --- | --- |
| FASTACT (2009) [[13](#_ENREF_13)] | Yes | Yes | Yes | NA | Yes | Yes |
| FASTACT-II (2013) [[14](#_ENREF_14)] | Yes | Yes | Yes | Yes | Yes | Yes |
| INTACT 1 (2004) [[7](#_ENREF_7)] | Yes | Yes | Yes | NA | Yes | NA |
| INTACT 2 (2004) [[8](#_ENREF_8)] | Yes | Yes | Yes | NA | Yes | Yes |
| TALENT (2007) [[9](#_ENREF_9)] | Yes | Yes | Yes | Yes | NA | Yes |
| TRIBUTE (2005) [[10](#_ENREF_10)] | Yes | Yes | Yes | NA | Yes | NA |
| CALGB30406(2012) [[12](#_ENREF_12)] | Yes | Yes | NA | Yes | Yes | Yes |
| Hirsch et al (2011) [[11](#_ENREF_11)] | Yes | Yes | NA | Yes | No | Yes |

NOTE: NA = not available
